# The Role of Estrogen Receptor–Targeted PET with 16α-^18^F-Fluoro-17β-Estradiol in Predicting Response to Endocrine Therapies in Metastatic Breast Cancer: A Metaanalysis

**DOI:** 10.2967/jnumed.125.270763

**Published:** 2026-01

**Authors:** Jennifer M. Specht, Jasper J.L. van Geel, Shaoli Song, Cheng Liu, Daniel S. Hippe, Nicholas A. DiGregorio, Christine J. Brand, Hannah M. Linden

**Affiliations:** 1 Division of Medical Oncology, Fred Hutchinson Cancer Center Clinical Research Division, Department of Medicine, University of Washington, Seattle, Washington;; 2 Department of Medical Oncology, University Medical Center Groningen, University of Groningen, Groningen, The Netherlands;; 3 Department of Nuclear Medicine, Fudan University Shanghai Cancer Center, Department of Oncology, Shanghai Medical College, Fudan University, Shanghai, China;; 4 Clinical Research Division, Fred Hutchinson Cancer Center, Seattle, Washington; and; 5 Medical Affairs, GE HealthCare, Chicago, Illinois

**Keywords:** radiopharmaceuticals, 16α-^18^F-fluoro-17β-estradiol, estrogen receptor, fluoroestradiol, metastatic breast cancer, PET imaging

## Abstract

[^18^F]16α-fluoro-17β-fluoroestradiol ([^18^F]FES) PET/CT imaging enables whole-body assessment of functional estrogen receptor (ER) expression in metastatic breast cancer (mBC). Identifying imaging biomarkers that predict endocrine therapy (ET) response remains a critical need in optimizing treatment selection. Our objective was to assess the predictive utility of [^18^F]FES PET/CT imaging in determining response to ET, with a focus on interlesional heterogeneity and individual patient outcomes. **Methods:** A systematic literature review and metaanalysis were conducted using 6 major databases through April 2024. Ten studies met inclusion criteria based on quantitative SUV reporting, use of FES PET/CT in mBC, and correlation with clinical outcomes. All patients had ER-positive mBC and received ET. Primary endpoints included progression-free survival (PFS) and response to ET. Patients were stratified by baseline [^18^F]FES PET/CT SUV_mean_ or SUV_max_ thresholds (including 1.8) and by interlesional [^18^F]FES heterogeneity (presence of both [^18^F]FES-positive and [^18^F]FES-negative lesions). **Results:** Responders had a significantly higher baseline SUV_mean_ than nonresponders (standardized mean difference, 0.91; 95% CI, 0.49–1.34; *P* < 0.001). Patients with a baseline SUV_max_ below 1.5 were significantly less likely to respond (odds ratio, 0.11; 95% CI, 0.02–0.72; *P* = 0.02). Across 5 studies, patients with heterogeneous [^18^F]FES uptake had a shorter median PFS (2.4–12.4 mo) than did those with all [^18^F]FES-positive lesions (14.6–23.6 mo), a statistically significant difference (ratio of median PFS, 0.25; 95% CI, 0.17–0.36; *P* < 0.001). In an individual-level analysis (*n* = 101), lesion-level [^18^F]FES-heterogeneous uptake was associated with a PFS of 5.5 versus 21.6 mo and a hazard ratio of 5.4 (95% CI, 3.2–9.4; *P* < 0.001). An [^18^F]FES SUV_max_ threshold of at least 1.8 was more prognostic of PFS than were higher SUV_max_ thresholds. **Conclusion:** [^18^F]FES PET/CT imaging provides prognostic insight beyond static ER testing by identifying functional heterogeneity in mBC. Lesion-level FES heterogeneity based on an SUV_max_ threshold of 1.8 may help stratify patients unlikely to benefit from ET, guiding more personalized treatment strategies.

## Visual Abstract

**Figure:**
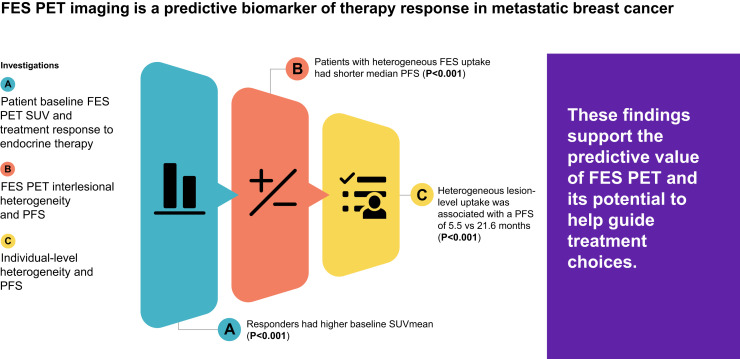

Breast cancer, the most common noncutaneous cancer in women throughout the world, remains challenging to manage, especially in the advanced stages, despite improved clinical outcomes due to an evolving treatment paradigm (1,2). Estrogen plays an important role in driving disease progression, and 70%–80% of BCs are estrogen receptor (ER)–positive (3,4). Of patients with new breast cancer diagnoses, approximately 5%–10% present with metastatic breast cancer (mBC), and nearly 30% of patients will progress to mBC over the course of their disease, with this risk being particularly pronounced in ER-positive tumors (2,3,5,6). In 2025, an estimated 170,000 women in the United States are living with mBC (7), and approximately 60% of metastatic breast tumors are ER-positive (8).Therefore, a biopsy to determine ER status is recommended at diagnosis, first recurrence, or instances of metastatic disease (9).

Although most ER-positive tumors initially respond to endocrine therapy (ET) (4,10), approximately 15%–20% demonstrate intrinsic resistance, and an additional 30%–40% develop acquired resistance, leading to progression in the metastatic disease (8). Consequently, ET becomes ineffective or fails to offer benefit for nearly half of patients with ER-positive mBC (11,12). When second-line ET is evaluated in patients with advanced disease, clinical benefit rates are even lower, with median progression-free survival (PFS) ranging from 2 to 8 mo (13–15). In addition, ER status may change over time, possibly in response to adjuvant therapies, with important implications for survival (16). A metaanalysis reported that 22.5% of distant metastases convert from ER-positive to ER-negative during therapy, whereas 21.5% convert from ER-negative to ER-positive, highlighting the importance of reassessing receptor status throughout disease progression (17).

Many strategies have been explored to overcome ET resistance. Epigenetic drugs, such as histone deacetylase inhibitors and DNA hypomethylation agents, are being investigated for their ability to reactivate ER expression and resensitize breast cancer cells and tumors to ET (18,19). Resistance may also occur through activation of alternative survival pathways, and combining ET with targeted inhibitors of cyclin-dependent kinase 4/6, phosphoinositide 3-kinase, human epidermal growth factor receptor 2, or fibroblast growth factor receptor signaling can potentially restore ER dependence, thereby improving sensitivity to ET (20).

Currently, there is a significant challenge in the management of patients with ER-positive mBC after initial therapy, as many may undergo treatments that provide minimal therapeutic gain, contributing to unnecessary toxicity, diminished quality of life, and increased health care costs (21,22). A better understanding of ER status and its predictive role in therapy response is critical to refining patient selection, avoiding futile treatments, and ultimately improving disease outcomes (23). Integrating precision approaches, such as dynamic assessments of ER functionality or resistance mechanisms, has the potential to optimize therapeutic outcomes while simultaneously reducing treatment burdens.

The effectiveness of ET depends on functional ER expression in ER-positive breast cancer lesions, and categorizing patients on the basis of functional status of ER may allow for prediction of response when assessing appropriate treatment strategies (10). Immunohistochemical analysis is the gold standard method to determine ER status in patients with mBC; however, it is limited because it identifies in a small sample of tissue only whether the ER is present and cannot determine the degree of functionality (24,25). Because of the spatial and temporal heterogeneity of ER expression, tissue-sampling techniques do not fully capture expression in all sites of disease. Quantitative determination of ER potential to functionally bind estradiol across the whole body may provide important guidance on potential response to ETs that may reduce therapeutic and financial burden and avoid futile treatments (25,26).

[^18^F]16α-fluoro-17β-fluoroestradiol ([^18^F]FES) is a radiolabeled form of estrogen that binds to functional ER and enables whole-body imaging via PET, without the need for biopsy. [^18^F]FES uptake, as measured by SUV on PET imaging, can provide information on the distribution and heterogeneity of ER binding as well as inform on the potential for ET to bind at the individual-lesion level (26,27). Although [^18^F]FES PET is often described as a functional imaging tool, it measures ER abundance and reflects ligand-accessible ER binding-site availability without giving direct evidence of whether the ER is functional within the tumor (28). Clinical studies have shown that even low levels of ER expression may be associated with survival benefits (29,30). Cumulative evidence has indicated that [^18^F]FES uptake in lesions may correlate with treatment response to ET in patients with mBC and therefore may have the potential to predict long-term clinical outcomes, such as PFS (30–32). Whole-body assessment of [^18^F]FES uptake may help overcome current limitations associated with ER quantification, such as the presence of nonfunctional ER, intrapatient heterogeneity within and between lesions in individual patients, changes in marker expression over time, and tissue-sampling limitations (9,10).

The Society of Nuclear Medicine and Molecular Imaging (SNMMI) convened a work group in 2021 to provide recommendations on clinical scenarios in which [^18^F]FES would have a meaningful impact on the management of ER-positive breast cancer (26). Although the work group reviewed studies to inform development of the appropriate-use criteria (AUC), a systematic aggregation and analysis of data from these studies was not performed.

The aim of this metaanalysis was to build on the AUC findings, determine the relationship between [^18^F]FES uptake in mBC lesions and therapy response to ET, and determine the relationship between lesion-level heterogeneity (presence in a single patient of both [^18^F]FES-positive [FES+] and [^18^F]FES-negative [FES−] lesions) and long-term outcomes after ET.

## MATERIALS AND METHODS

### Literature Search

This systematic review was registered with the International Prospective Register of Systematic Reviews (PROSPERO; identification no. CRD1135213). To assess the predictive value of [^18^F]FES PET/CT for ET response in breast cancer, a comprehensive search was conducted across PubMed, MEDLINE, Embase, Web of Science, Cochrane Library, and Emcare through April 2024. Search strategies were adapted from appendix D of the SNMMI’s AUC for [^18^F]FES PET. Keywords included “^18^F-FES PET,” “breast cancer,” “endocrine therapy response,” “SUV_max_,” “SUV_mean_,” “estrogen receptor imaging,” and “lesion heterogeneity,” among others, to capture relevant studies in mBC. Institutional Review Board approval was not required for this metaanalysis, as the study involved the analysis of previously published data and did not include any direct research involving human subjects.

### Study Selection

Study selection followed the SNMMI AUC protocol, applying a modified Delphi process and the PICOTS (patient population, intervention, comparator, outcome, time, and setting) framework to define eligibility. Included studies met the following criteria: they involved advanced or metastatic ER-positive breast cancer, they assessed [^18^F]FES PET/CT using SUV_max_ or SUV_mean_, and they correlated imaging findings with clinical outcomes such as PFS or endocrine response. Only full-text, English-language, human studies were considered. Excluded were studies without quantitative [^18^F]FES data, those focused on nonmetastatic disease or other cancers, reviews, editorials, case reports, conference abstracts, and studies using nonstandard imaging protocols. Two reviewers independently screened and extracted data for analysis. Study quality was assessed using the Newcastle–Ottawa Scale for cohort studies (33).

### Investigations and Outcome Measures

**FIGURE 1.**
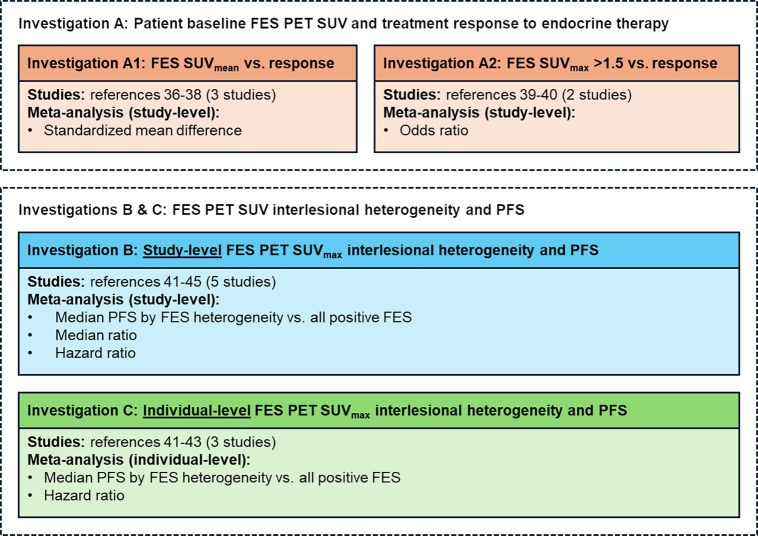
Investigations on relationships between [^18^F]FES PET SUV and response to ET or PFS.

Three investigations were conducted to explore the associations among patient baseline [^18^F]FES PET SUV and treatment response to ET (investigation A), [^18^F]FES PET interlesional heterogeneity and PFS (investigation B), and individual-level heterogeneity and PFS (investigation C) (Fig. 1).

In investigation A, response to therapy was a binary measure of response across all lesions or up to 7 of the most intense lesions or a measurable improvement in lesion uptake (e.g., an increase in SUV_max_ or SUV_mean_) on follow-up scans, correlated with stable disease, partial response, or complete response based on RECIST or no response (defined as progressive disease according to RECIST or clinical progression). This investigation was divided into 2 subinvestigations (A1 and A2) because the studies included for each analysis reported results relating to [^18^F]FES uptake and response in ways that could not be combined because of differences in quantitative measurements. In investigation A1, the corresponding studies analyzed baseline [^18^F]FES SUV_mean_ as a continuous variable stratified by objective response; in investigation A2, the corresponding studies dichotomized baseline SUV_max_ as [^18^F]FES SUV greater than 1.5 versus less than 1.5 and summarized its association with objective response using the odds ratio.

In investigation B, study-level data were analyzed to examine the relationship between interlesional heterogeneity and PFS. Lesions were defined on the basis of uptake identified on [^18^F]FES PET, with or without anatomic correlation from companion imaging modalities (e.g., CT, MRI, [^18^F]FDG PET, or bone scanning) as reported in the original studies. Interlesional heterogeneity was qualitatively defined as patients displaying both FES+ and FES− lesions compared with patients with all FES+ lesions. PFS was defined as the time between the date of starting ET and progressive disease or death from any cause. PFS was censored at the last follow-up if the patient was alive and progression-free.

In investigation C, individual-level data from multiple studies were combined and analyzed to further examine the relationship between interlesional heterogeneity and PFS. A lesion with an SUV_max_ of at least 1.8 was considered FES+, lesions with an SUV_max_ of less than 1.8 were considered FES−, and [^18^F]FES heterogeneity was defined as the presence of at least 1 FES− lesion within the disease burden of an individual patient, as in investigation B. Median PFS was compared between patients with [^18^F]FES-heterogeneous and -homogeneous (all FES+) lesions.

### Statistical Analysis

For investigations A and B, study-level metaanalyses used fixed-effects models (FEMs) to calculate pooled standardized mean differences, odds ratios, hazard ratios (HRs), mPFS, and ratios of medians, with pooling performed on the log scale (Fig. 1). The δ-method was applied when needed to transform CI, and for 1 study with an unreported upper bound, the lower bound was used assuming CI symmetry. Random-effects models (REMs) were used in sensitivity analyses to account for between-study heterogeneity. Heterogeneity was assessed using the Cochrane Q and the I^2^ statistic. Risk of publication bias was assessed using funnel plots and the rank correlation test (34). This assessment was limited to investigation B (5 studies) because of the lower number of studies available for investigation A1 (3 studies) and investigation A2 (2 studies).

For investigation C (3 studies), individual-level data were merged across studies. mPFS was estimated using Kaplan–Meier methods and compared between [^18^F]FES-heterogeneous and -homogeneous subgroups via Cox proportional hazards models. Alternative SUV_max_ thresholds (1.8–4.0, in 0.1 increments) were explored to assess their impact on heterogeneity classification and HRs, visualized with Kaplan–Meier curves and HR plots. All *P* values were 2-sided, with significance defined as a *P* value of less than 0.05.

## RESULTS

### Study Characteristics

**FIGURE 2.**
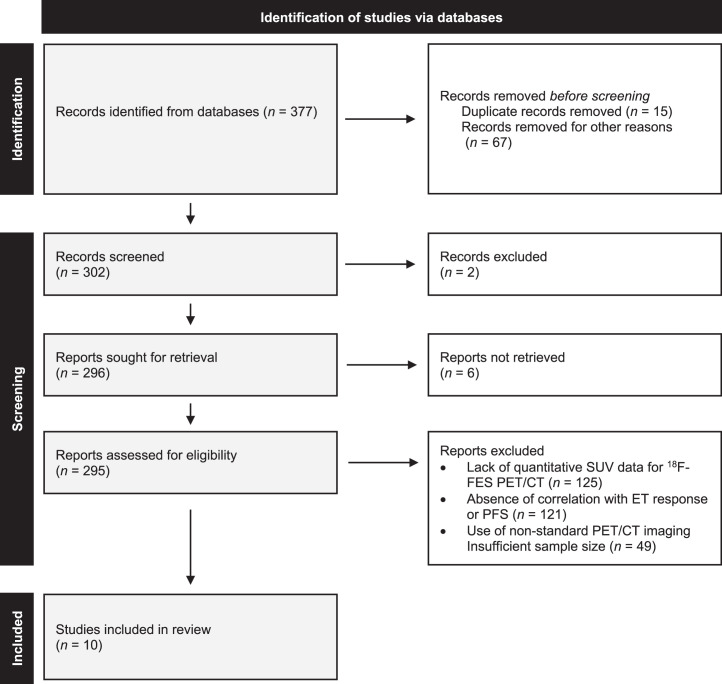
PRISMA (Preferred Reporting Items for Systematic Reviews and Meta-Analyses) flowchart of screened and included studies.

In total, 377 records were identified, of which 10 studies (469 patients) met the eligibility criteria (Supplemental Table 1; supplemental materials are available at http://jnm.snmjournals.org). Studies were excluded primarily because of lack of quantitative [^18^F]FES SUV data, lack of correlation with ET response or PFS, nonmetastatic patient populations, or use of nonstandard imaging protocols. The study selection process is summarized in Figure 2.

Of the 10 eligible studies, 5 could be included in investigation A comparing baseline [^18^F]FES SUV and response. The 5 remaining studies reported comparable [^18^F]FES SUV and PFS measures and could be included in investigation B. Of these 5, individual-level data obtainable from 3 studies were included in investigation C. All studies were rated as of high quality across the domains of selection, comparability, and outcome (Supplemental Table 2). Detailed ratings for each item are shown in Supplemental Table 3. The included studies reported imaging start times between 50 and 90 min after injection, which falls within the recommended diagnostic imaging time–activity curve window for [^18^F]FES (Supplemental Table 4) (35).

### Baseline [^18^F]FES PET SUV and Treatment Response (Investigation A)

Initial pooled analyses from 3 studies (102 patients) demonstrated that patients who responded to ET had a significantly higher baseline SUV_mean_ than did nonresponders (standardized mean difference, 0.91; 95% CI, 0.49–1.34; *P* < 0.001) (Supplemental Fig. 1) (36–38). These findings were consistent across FEMs and REMs. For investigation A2, analyses of 2 different studies (*n* = 62) revealed that patients with a baseline SUV_max_ below 1.5 were significantly less likely to respond to ET (odds ratio, 0.11; 95% CI, 0.02–0.72; *P* = 0.02) (Supplemental Fig. 2) (39,40).

### [^18^F]FES PET Interlesional Heterogeneity and PFS (Investigation B)

Five studies were applicable to investigation B (41–45). Across these studies, there was variation in which lesions were analyzed and how FES+ was defined. Studies analyzed all lesions (≤20 per patient) (41,42), all nonbone lesions and up to 5 bone lesions per skeletal region per patient (43), the 3 largest lesions (44), or all identified lesions (45). FES+ was defined as an SUV_max_ of at least 1.8 in 3 studies (41–43) and as an SUV_max_ of at least 2.0 in 2 studies (44,45). Patients were grouped by whether all analyzed lesions were FES+ or whether there was a mixture of FES+ and FES− lesions ([^18^F]FES-heterogeneous group). The analysis of [^18^F]FES interlesional heterogeneity across the 5 studies (64 patients in the [^18^F]FES-heterogeneous groups) showed that patients with both FES+ and FES− lesions had a consistently shorter mPFS than did those with all FES+ lesions. mPFS in the [^18^F]FES-heterogeneous group ranged from 2.4 to 12.4 mo (pooled, 4.9 [FEM]–5.3 [REM]), whereas the FES+ homogeneous group demonstrated an extended PFS of 14.6–23.6 mo (pooled, 18.9 [FEM]–18.8 [REM]) (Table 1). These differences were statistically significant based on the ratio of mPFS (FEM, 0.25; 95% CI, 0.17–0.36; *P* < 0.001) and the HR (FEM, 4.6; 95% CI, 1.7–14.9; *P* < 0.001). There was some between-study variability in these ratios (*P* = 0.010), but the REM-based estimates were similar to the FEM estimates (Table 1). Funnel plots were used to assess study heterogeneity and potential for publication bias (Supplemental Fig. 3). No evidence of publication bias was found, though this assessment was limited to only 5 studies available (rank test *P* = 0.23–0.82).

**TABLE 1.** Progression-Free Survival by [^18^F]FES PET Heterogeneity in Patients with ER+ mBC (Investigation B)

	Median PFS		[^18^F]FES heterogeneity vs. all FES+	
Parameter	[^18^F]FES heterogeneity*	All FES+^†^	Median PFS ratio	HR
Studies included				
Liu et al. 2020 (41) (*n* = 35)	5.5 (2.3–8.7), *n* = 12	18.4 (12.2–29.8), *n* = 23	0.30 (0.14–0.62)	6.6 (2.6–17.0)
He et al. 2020 (43) (*n* = 36)	7.2 (3.0–11.4), *n* = 10	14.6 (8.4–20.8), *n* = 26	0.49 (0.24–1.01)	2.3 (0.9–5.9)
Boers et al. 2020 (45) (*n* = 27)	6.2 (3.2–11.3), *n* = 20	16.8 (4.8–∞), *n* = 7	0.37 (0.09–1.49)	2.1^‡^ (0.8–6.9)
Liu et al. 2022 (42) (*n* = 56)	2.4 (1.1–3.7), *n* = 10	23.6 (15.8–31.4), *n* = 46	0.10 (0.05–0.19)	25.0 (7.7–100.0)
Gennari et al. 2024 (44) (*n* = 125)^§^	12.4 (3.1–59.6), *n* = 12	18.0 (11.2–23.1), *n* = 113	0.69 (0.15–3.16)	—
Pooled estimates				
Fixed-effects model	4.9 (3.7–6.6)	18.9 (15.7–22.8)	0.25 (0.17–0.36)	4.6 (2.7–7.7)
Random-effects model	5.3 (3.2–8.5)	18.8 (15.4–23.0)	0.29 (0.15–0.59)	5.1 (1.7–14.9)
Heterogeneity I^2^	61%	0%	67%	76%
Cochron Q test	*P* = 0.032	*P* = 0.51	*P* = 0.010	0.010

* Patient with both FES+ and FES− lesions.

† Patients with FES+ lesions only.

‡ Patients with [^18^F]FES heterogeneity (*n* = 20) and FES− lesions only (*n* = 3) were combined in HR calculation in original study.

§ Patients with average [^18^F]FES SUV_max_ ≥ 2 received single-agent ET until progressive disease (registered arm). Patients with average [^18^F]FES SUV_max_ < 2 were randomized to single-agent ET (arm A) or chemotherapy (arm B). All patients were ER-positive, most with >50% staining. Registered arm was used as all-FES+ group and arm A as [^18^F]FES-heterogeneous group; these groupings are approximate as study did not provide enough detail to confirm [^18^F]FES status of each lesion. Arm B (*n* = 16) was not included in metaanalysis because of non-ET; median PFS was 23.0 mo (95% CI, 7.7–30.0) in arm B.

Data in parentheses are 95% CIs.

### [^18^F]FES PET Individual-Level Heterogeneity and PFS (Investigation C)

The individual-level data from 3 studies eligible for investigation C were merged (Supplemental Fig. 4), yielding a cohort of 101 patients with at least 1 FES+ lesion ([^18^F]FES SUV_max_ ≥ 1.8) and 878 individual [^18^F]FES–quantified lesions (median lesions per patient, 7; interquartile range, 4–12) (41–43). As noted in investigation B, all lesions (up to 20 per patient) (41,42) or all nonbone lesions and up to 5 bone lesions per region were included (43). Based on lesion site, 40 patients had visceral metastases (24 with bone metastases as well and 16 without), 47 had bone metastases without any visceral metastases (31 with both bone and soft-tissue lesions and 16 with only bone lesions), and 14 had soft-tissue lesions only. Overall, 71 had at least 1 bone lesion, 76 had at least 1 lymph node lesion, 25 had at least 1 lung lesion, and 33 had at least 1 lesion of any other type (Supplemental Table 5).

**FIGURE 3.**
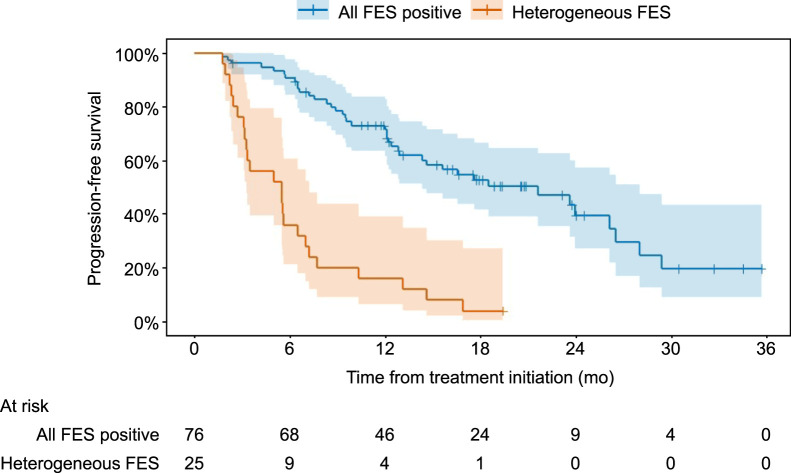
Progression-free survival stratified by [^18^F]FES PET heterogeneity (investigation C).

Of the 101 patients, 25 were [^18^F]FES-heterogeneous, with at least 1 FES− lesion, whereas the remaining 76 patients had only FES+ lesions. Across the 878 lesions from all patients, the median [^18^F]FES SUV_max_ was 6.0 (interquartile range, 3.9–9.4) (Supplemental Table 6). Overall, 64 patients experienced progression or death during follow-up, with a mPFS of 13.1 mo (95% CI, 10.3–21.6 mo). Median PFS was significantly shorter in the [^18^F]FES-heterogeneous group than in the [^18^F]FES-homogeneous group (5.5 [95% CI, 3.3–7.2] vs. 21.6 mo [95% CI, 14.3–28.0]; HR, 5.4 [95% CI, 3.2–9.4]; *P* < 0.001), supporting the prognostic value of lesion-level [^18^F]FES heterogeneity (Fig. 3).

**FIGURE 4.**
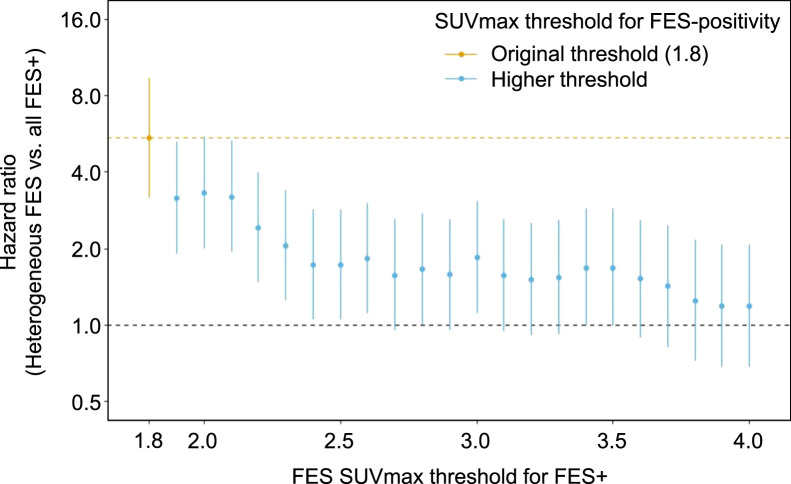
Effect of SUV_max_ threshold on PFS HR for [^18^F]FES heterogeneity (investigation C).

Alternative [^18^F]FES SUV_max_ thresholds for [^18^F]FES positivity were explored to determine whether risk stratification by [^18^F]FES status could be further optimized. When the [^18^F]FES-positivity threshold was increased above the original threshold of 1.8 up to 4.0, the HR for heterogeneous versus homogeneous numerically decreased, ranging from 1.2 (95% CI, 0.7–2.1; *P* = 0.55), with a threshold of at least 4.0, to 3.3 (95% CI, 2.0–5.5; *P* < 0.001), with a threshold of at least 2.0 (Fig. 4). In other words, over the range of thresholds from 1.9 to 4.0, none of the resulting HRs were higher than the HR based on the original threshold of 1.8. This weakening of the HR with increasing [^18^F]FES-positivity threshold was driven by improvements in the PFS of the [^18^F]FES-heterogeneous group (a higher positivity threshold leads to more patients being classified as heterogeneous), whereas the PFS of the all-FES+ group was stable across [^18^F]FES-positivity thresholds (Fig. 4). This pattern suggests a threshold at an [^18^F]FES SUV_max_ of 1.8, with outcomes improving markedly above this level but higher uptake beyond 1.8 offering little additional benefit.

## DISCUSSION

This metaanalysis reviewed published studies to determine the effectiveness of [^18^F]FES PET in predicting response to ET in patients with mBC. Although higher baseline [^18^F]FES uptake was associated with ET response, the most compelling finding emerged from the analysis of interlesional heterogeneity. Specifically, patients with heterogeneous [^18^F]FES uptake (defined as a mix of FES+ and FES− lesions) consistently experienced shorter PFS than those with homogeneous FES+ disease. Across the studies that evaluated individual-level [^18^F]FES heterogeneity versus homogeneity, the [^18^F]FES-homogeneous group demonstrated a substantially longer PFS. This consistent pattern across diverse study designs and populations strongly supports the prognostic relevance of lesion-level heterogeneity as measured by [^18^F]FES PET when most subjects were receiving first-line ET for mBC.

These findings suggest that interlesional [^18^F]FES heterogeneity is not only a surrogate marker of endocrine resistance but also a practical imaging biomarker that could be integrated into clinical workflows. Identifying patients with ER heterogeneity may help clinicians anticipate a suboptimal response to ET and consider alternative strategies, such as combination regimens or earlier transition to non–endocrine-based therapies. The stratification of patients on the basis of this imaging phenotype represents a significant advance in functional precision oncology.

The distribution of metastatic sites in investigation C was consistent with typical patterns of ER-positive mBC, with bone and nodes often involved (46,47). Bone lesions are relatively difficult to biopsy (48), suggesting the utility of [^18^F]FES PET for assessing heterogeneity at the individual level. The inclusion of patients with visceral disease may provide context for the association between uptake heterogeneity and shorter PFS, since visceral involvement is generally linked to less favorable outcomes (49).

An [^18^F]FES SUV_max_ of at least 1.8 was applied in our individual-level analysis as a threshold to indicate a positive lesion. Although there is no universally accepted standard, prior studies have used [^18^F]FES SUV_max_ thresholds ranging from 1.5 to 2.0. Some reports note that background uptake in normal tissues can exceed 1.5 and that responders to ET may still present with a tumor SUV_max_ below 2.0 (50). In this context, intermediate thresholds such as 1.8 may represent a clinically relevant midpoint—balancing sensitivity and specificity—by reducing false positives associated with lower cutoffs (e.g., 1.5) while avoiding underestimation of responsive patients when using higher thresholds (e.g., 2.0) (51,52). Although lower thresholds correspond better to ER immunohistochemistry (53), this is a comparison different from that of ER functionality; therefore, it is not unreasonable to assume that if potential false-positive findings are decreased by increasing the threshold, the response prediction improves. Furthermore, we found that [^18^F]FES SUV_max_ thresholds of 1.9–4.0 were less predictive of PFS than was an SUV_max_ of at least 1.8, as higher thresholds tended to underestimate responsiveness to ET (i.e., false negatives). Although our findings better support the use of an SUV_max_ threshold of at least 1.8, we cannot rule out that lower thresholds (e.g., 1.5–1.7) may be even more predictive of PFS. Additional standardization strategies, including PET/CT scanner calibration, body mass index adjustment, and SUV normalization techniques, may further refine the clinical utility of [^18^F]FES PET (10).

This study provides systematic evidence that supports several clinical scenarios described in the AUC publication from the SNMMI (25). In particular, the association between [^18^F]FES uptake heterogeneity and shorter PFS supports the use of [^18^F]FES PET after progression when considering another line of ET and at the time of metastatic diagnosis when planning ET. Our data add evidence that complements and strengthens the AUC recommendations while further supporting broader clinical adoption of [^18^F]FES PET in practice.

### Limitations

This metaanalysis has several limitations. Heterogeneity in study design, patient populations, and imaging protocols may limit generalizability, and the predominance of observational studies restricts causal inference. Multiple studies from the same institution, small sample sizes, potential cohort overlap, and unmeasured prior therapies may introduce bias or increase variability. Most studies evaluated endocrine monotherapy, with limited inclusion of cyclin-dependent kinase 4/6 inhibitors, and none involved mammalian target of rapamycin or phosphatidylinositol-4,5-bisphosphate 3-kinase catalytic subunit alpha-targeted agents, reducing applicability to current combination regimens.

The lack of standardized SUV thresholds further complicates clinical translation. Although both investigation A1 and investigation A2 explored associations between [^18^F]FES SUV and response, the 2 groups of studies reported results in different ways—with [^18^F]FES SUV_mean_ as a continuous variable versus [^18^F]FES SUV_max_ as a dichotomized variable—preventing pooling into a more uniform analysis. However, when considered together, investigations A1 and A2 provide evidence for a relatively large association between baseline [^18^F]FES SUV and response to ET. In investigation C (individual-level metaanalysis), all 3 studies came from the same institution, and thresholds below 1.8 could not be analyzed because of missing data. Nonetheless, consistent and clinically meaningful PFS differences between [^18^F]FES-heterogeneous and -homogeneous groups across studies support further investigation of [^18^F]FES PET–based heterogeneity in future trials and clinical decision-making for ER-positive mBC.

### Future Research and Gaps

Although this metaanalysis highlights the potential of [^18^F]FES PET as a predictive biomarker for ET response in mBC, several gaps remain that warrant further investigation. First, future studies should focus on standardizing SUV thresholds for [^18^F]FES PET imaging to improve its predictive accuracy across diverse patient populations and clinical settings. Larger, prospective trials are needed to validate these thresholds and assess their clinical utility.

Moreover, the applicability of [^18^F]FES PET across different lines of therapy remains uncertain. In earlier lines, in which functional ER expression is generally preserved, [^18^F]FES could improve patient selection by identifying those likely to benefit from endocrine monotherapy or combination therapies, potentially delaying resistance and progression. Prospective trials evaluating [^18^F]FES across the disease course, along with standardized imaging protocols and integration with molecular biomarkers, are needed to optimize its role in guiding personalized treatment, improving outcomes, and minimizing futile therapies.

Finally, the integration of automated tools for heterogeneity analysis, together with [^18^F]FES PET with complementary imaging techniques such as [^18^F]FDG PET, may provide a more comprehensive assessment of tumor biology and further strengthen the role of [^18^F]FES PET in clinical decision-making.

## CONCLUSION

Assessment of receptor status remains a cornerstone of mBC management, with biopsy recommended to guide treatment selection. However, our metaanalysis highlights that [^18^F]FES PET offers unique clinical value by enabling noninvasive, whole-body evaluation of functional ER expression. Most notably, the presence of interlesional heterogeneity—identified through mixed FES+ and FES− lesions—was consistently associated with significantly shorter PFS across multiple studies. These findings support the integration of [^18^F]FES PET not only for assessing the potential binding ability of ET at the lesion level but also for stratifying patients by likelihood of ET benefit. By identifying those less likely to respond, [^18^F]FES PET has the potential to refine therapeutic decision-making, reduce unnecessary treatment, and improve clinical outcomes in mBC.

## DISCLOSURE

Jennifer Specht, Daniel Hippe, and Hannah Linden are consultants for GE HealthCare. Nicholas DiGregorio and Christine Brand are salaried employees of GE HealthCare. No other potential conflict of interest relevant to this article was reported.

## Notes

KEY POINTS **QUESTION:** Can [^18^F]FES PET imaging predict response to ET in patients with ER–positive mBC? **PERTINENT FINDINGS:** In this metaanalysis of 10 studies (*n* = 469), higher baseline [^18^F]FES uptake was associated with response to ET, whereas patients with both FES+ and FES− lesions had a significantly shorter PFS than did patients with only FES+ lesions. Lesion-level heterogeneity based on an SUV_max_ threshold of 1.8 was the strongest predictor of poor outcomes. **IMPLICATIONS FOR PATIENT CARE:** Identifying [^18^F]FES heterogeneity with PET imaging may help personalize treatment by identifying patients less likely to benefit from ET.
